# A Narrative Review on the Role of Dalbavancin in the Treatment of Bone and Joint Infections

**DOI:** 10.3390/antibiotics12101492

**Published:** 2023-09-28

**Authors:** Dimitra Dimopoulou, Elpis Mantadakis, Christos Koutserimpas, George Samonis

**Affiliations:** 1 Second Department of Pediatrics, “Aghia Sophia” Children’s Hospital, 115 27 Athens, Greece; dimi_med@hotmail.com; 2 Department of Pediatrics, University General Hospital of Alexandroupolis, 681 00 Alexandroupolis, Greece; emantada@med.duth.gr; 3 Department of Orthopaedics and Traumatology, “251” Hellenic Air Force General Hospital of Athens, 115 25 Athens, Greece; chrisku91@hotmail.com; 4 Department of Medicine, University of Crete, 715 00 Heraklion, Greece; 5 First Department of Medical Oncology, “Metropolitan” Hospital, 185 47 Attica, Greece

**Keywords:** dalbavancin, bone and joint infections, osteomyelitis, septic arthritis, spondylodiscitis, diabetic foot infection, prosthetic joint infection

## Abstract

Bone and joint infections (BJI) require prolonged antimicrobial treatment, leading to lengthy hospitalizations, high costs, the risk of nosocomial infections, and the development of antimicrobial resistance. Dalbavancin is a novel semisynthetic lipoglycopeptide approved for the treatment of adults and children with acute bacterial skin and skin structure infections. This narrative review aims to summarize the characteristics of dalbavancin and the current scientific evidence regarding its clinical efficacy and safety in the treatment of BJI. A literature search until June 2023 was performed to identify all published research about the role of dalbavancin in the management of BJI. Due to its unique pharmacokinetics characterized by prolonged half-life, high bactericidal activity against most Gram-positive bacteria, a good safety profile, and high tissue penetration, dalbavancin can be a valuable alternative to the treatment of BJI. Clinical studies have shown its non-inferiority compared to conventional therapies in BJI, offering potent activity against key pathogens and an extended dosing interval that may shorten hospitalization. In conclusion, dalbavancin represents a promising treatment option for BJI with a favorable safety profile, but further research in both adults and particularly children, who are ideal candidates for long-acting antibiotics, is necessary to evaluate the role of dalbavancin in BJI.

## 1. Introduction

Bone and joint infections (BJI), including osteomyelitis, septic arthritis, spondylodiscitis, and prosthetic joint infection (PJI), are inflammatory processes of the bone and synovial joint characterized by progressive destruction of the involved tissues, usually caused by pyogenic microorganisms and rarely by Mycobacteria and fungi [1,2,3,4]. The pathogenesis of BJI is related to either hematogenous dissemination or direct inoculation of the involved bacterial pathogens as a result of trauma or infection from contiguous tissues [1,2,3,4]. Gram-positive bacteria are the most prevalent, with *Staphylococcus aureus* being the most frequently identified [5]. The management of BJI is challenging due to the poor vascularity of the bones and synovial joints and is associated with a significant burden to healthcare systems, as these infections require prolonged courses of antimicrobial treatment [5]. Current guidelines recommend at least six weeks of parenteral antimicrobial therapy as the standard treatment regimen for bone infections caused by Gram-positive microorganisms, along with surgical drainage or debridement [6,7,8]. However, an open-label, randomized, controlled, non-inferiority trial comparing 6 with 12 weeks of antibiotic therapy in patients with microbiologically confirmed PJI demonstrated that antibiotic therapy for 6 weeks resulted in a higher percentage of patients with unfavorable outcomes. As a result, a 12-week course of antibiotics was recommended for these infections [9]. This prolonged antimicrobial treatment results in long hospitalizations, high costs, and rates of nosocomial infections and antimicrobial resistance [5].

The emergence of multidrug-resistant Gram-positive microorganisms has led to an increased length of hospital stay, healthcare costs, morbidity, and mortality. Therefore, there is a clear need for the development of new antimicrobial agents against these microorganisms [10]. More specifically, methicillin-resistant *S. aureus* (MRSA) has become a challenging public health problem across the United States and Europe [11]. Vancomycin is the antimicrobial of choice for MRSA osteoarticular infections, even though it penetrates bones poorly. It requires careful monitoring of drug levels to ensure safety and efficacy and is potentially nephrotoxic. Another limitation of the use of vancomycin for the treatment of BJI is its moderate activity against biofilms [12]. Moreover, its increased use worldwide has led to a rising incidence of vancomycin-resistant *S. aureus* (VRSA) isolates [13]. Daptomycin, a lipopeptide antibiotic, is an alternative therapy to vancomycin and is used for the treatment of infections caused by Gram-positive bacteria [14,15]. Daptomycin has an off-label clinical use for BJI, and it requires monitoring as it causes adverse effects such as myopathy and rhabdomyolysis. In contrast, prolonged use may result in *Clostridium difficile*-associated diarrhea and pseudomembranous colitis [14,15]. Also, linezolid, a synthetic oxazolidinone antimicrobial, is an alternative to glycopeptides to treat serious infections due to resistant Gram-positive organisms [16,17]. The use of this antibiotic in BJI is off-label. However, its prolonged use may result in myelosuppression and pseudomembranous colitis [16,17]. Thus, the increasing antimicrobial resistance of Gram-positive cocci, along with the need for prolonged antimicrobial administration for the treatment of BJI, has directed the focus of the scientific community toward the development of novel, effective, and safe alternative antimicrobials to vancomycin that can be used for outpatient parenteral antibiotic therapy (OPAT) [5,18,19].

Continuous efforts have led to the development of more potent semisynthetic glycopeptides, such as telavancin, oritavancin, and dalbavancin [5,18,19]. The latter is a novel second-generation, semisynthetic lipoglycopeptide approved by the European Medicines Agency (EMA) and the U.S. Food and Drug Administration (FDA) for the treatment of acute bacterial skin and skin structure infections in adults and children [20,21]. Due to its unique pharmacokinetic profile characterized by prolonged half-life, high bactericidal activity against most Gram-positive bacteria, a good safety profile, and high tissue penetration, dalbavancin can be a valuable alternative to prolonged daily in-hospital intravenous treatment or OPAT for severe deep-seated Gram-positive infections, such as BJI and endocarditis [22].

The purpose of this narrative review is to summarize the characteristics of dalbavancin and the current scientific evidence regarding its role in the treatment of BJI, as well as to present a comprehensive overview of the most recent data on the clinical efficacy and safety of this novel antimicrobial in the management of these difficult-to-treat infections.

## 2. Search Strategy

A literature search was performed to identify all published research, such as original articles, reviews, and systematic reviews/meta-analyses, using the keywords “dalbavancin”, “bone and joint infections”, “osteomyelitis”, “septic arthritis”, “spondylodiscitis”, “diabetic foot infection”, and “prosthetic joint infection”. Records were retrieved from PubMed/Medline until June 2023 without language restrictions. Reference lists of included articles were also screened to identify possible studies missed by the initial literature search.

## 3. Characteristics and Profile of Dalbavancin

### 3.1. Pharmacological and Pharmacokinetic Characteristics

**Figure 1:**
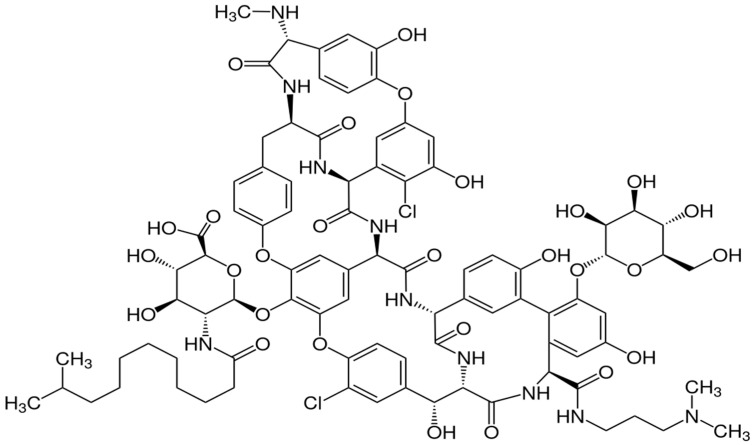
Chemical structure of dalbavancin.

Dalbavancin is a semisynthetic lipoglycopeptide derived from A40926, a naturally occurring glycopeptide produced by the actinomycete *Nonomuraea* species that has a comparable structure to teicoplanin (Figure 1) [23].

Its antimicrobial activity is characterized by the inhibition of cell wall synthesis and an anchoring mechanism, increasing its binding affinity to the target and its antimicrobial activity [23]. Its lipophilic side chain results in more rapid and effective bactericidal activity than vancomycin or teicoplanin and a prolonged half-life of approximately 14.5 days (346 h), allowing for weekly administration [23]. Dalbavancin is available only for intravenous administration and is characterized by slow absorption, high protein binding (93%), extensive tissue distribution, minimal metabolism, and non-renal elimination, contributing to its unique clinical advantages [24]. Serum concentrations do not change significantly in renal or hepatic impairment, and dose adjustment is needed only in patients with creatinine clearance <30 mL/min [25].

**Figure 2:**
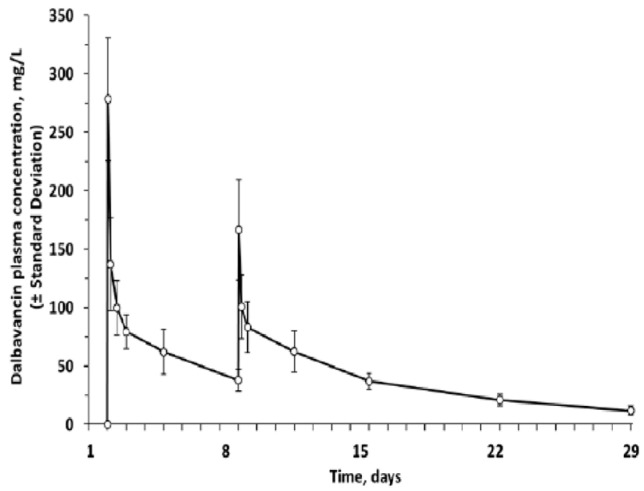
Dalbavancin mean plasma concentration (±SD) versus time (days) in healthy subjects following 30 min intravenous administration of 1000 mg dalbavancin on day 1 and 500 mg on day 8 (DALVANCE^®^ SPC). SD: standard deviation.

Dalbavancin exhibits linear, dose-dependent pharmacokinetics (Figure 2), and the pharmacokinetic/pharmacodynamic index that predicts in vivo efficacy is the ratio of the area under the curve to the minimum inhibitory concentration (AUC/MIC) [26].

Two dosing regimens have been evaluated for the treatment of BJI: a regimen of 1500 mg given on days 1 and 8, as well as a regimen of 1000 mg initially, followed by 4 subsequent weekly doses of 500 mg [27] (Table 1). Based on bone concentration–time profiles, intravenous administration of 1000 mg of dalbavancin on day 1, followed by 500 mg weekly for seven additional weeks, was well tolerated and did not demonstrate evidence of drug accumulation [27]. Also, dalbavancin concentrations in cortical bone 12 h after infusion of a single intravenous dose of 1000 mg were 6.3 μg/g and 2 weeks later remained high at 4.1 μg/g [27]. It has been shown that a two-dose, once-weekly regimen provides tissue exposure higher than the dalbavancin MIC for *S. aureus* for 8 weeks, and thus, it is the optimal approach for the treatment of BJI [27,28,29].

The pharmacokinetic properties of dalbavancin have also been examined in children. An open-label, multicenter, single-dose phase 1 study evaluated the pharmacokinetics of dalbavancin in hospitalized adolescents 12–17 years of age [30]. A single dose of 1000 mg of dalbavancin was administered as a 30-min intravenous infusion to subjects weighing >60 kg and 15 mg/kg for subjects weighing <60 kg [30]. The terminal half-life was approximately 9 days and was similar for dalbavancin dosages of 1000 mg and 15 mg/kg. Median dalbavancin plasma exposures (Cmax and AUC) were similar between the two groups and slightly lower than exposures in adults given 1000 mg [30]. Another phase 1 open-label, multicenter study investigated the pharmacokinetics of a single dose of intravenous dalbavancin in hospitalized children 3 months to 11 years of age [20]. The age-dependent dosing regimens that were found to achieve similar dalbavancin exposure to that in adults are described in Table 1 [20].

### 3.2. Antimicrobial Characteristics and Activity

Dalbavancin has bactericidal activity against *S. aureus*, including MRSA, as dalbavancin with a MIC90 of 0.06 mg/L has been demonstrated to be 16-fold more potent than daptomycin and 32-fold more potent than vancomycin and linezolid [31,32]. Dalbavancin is the most active agent against coagulase-negative staphylococci (CoNS) (MIC90 ≤ 0.06 mg/L) [31,32]. Furthermore, dalbavancin is 16-fold more potent against hemolytic streptococci (MICs90 0.03–0.047 mg/L) than vancomycin (MIC90 of 0.75 mg/L) [31,32]. All vancomycin-susceptible enterococci are inhibited (MIC90 ≤ 0.06 mg/L), but dalbavancin is not active against enterococci with the VanA phenotype (MIC90 > 4 mg/L) and is only partially active against VanB isolates [31,32]. All Gram-negative bacteria are resistant to dalbavancin, as it is not able to pass through the bacterial outer membrane [18]. Finally, dalbavancin exhibits potent antimicrobial in vitro and in vivo activity against biofilms caused by MRSA [33,34].

In surveillance testing over the decade 2002–2012 on 62,195 *S. aureus* isolates, dalbavancin was active against isolates that were non-susceptible to daptomycin, linezolid, or tigecycline. Overall, 99.8% of multidrug-resistant MRSA isolates were inhibited by dalbavancin at <0.12 g/mL (MIC50/90, 0.06/0.06 g/mL), the current U.S. FDA breakpoint. Overall, only 0.35% of the monitored *S. aureus* isolates were non-susceptible, i.e., had a dalbavancin MIC of either 0.25 or 0.5 g/mL [35].

### 3.3. Safety and Tolerability

The safety and tolerability of dalbavancin are generally favorable, with adverse events reported in a small percentage of patients [36]. Common adverse events, which are usually mild to moderate in severity, are described in Table 2 [36]. It is of note that patients treated with dalbavancin for skin and soft tissue infections had fewer adverse events compared to those treated with vancomycin or linezolid [37]. Although dalbavancin is characterized by a long half-life, late-onset adverse events were uncommon. In contrast to skin and soft tissue infections, BJI requires a longer duration of treatment, with multiple weekly doses. No accumulation of the drug and no serious adverse events were observed in patients who received extended courses of dalbavancin for BJI [27,38]. Drug–drug interactions with other medications are not frequent, as dalbavancin is not metabolized by the hepatic CYP450 system [23,36]. Similarly, the safety profile of children is comparable to that of adults [20,30]. Dalbavancin is well tolerated, and no serious or severe adverse effects associated with the treatment were described in the pediatric population. Also, no deaths or evidence of ototoxicity due to dalbavancin administration were observed [20,30].

### 3.4. Pharmacoeconomic Characteristics

Several studies have evaluated the cost-effectiveness of dalbavancin compared to other treatment options for various infections [39,40,41]. BJI correlates with a protracted hospital stay. The prolonged hospital length of stay leads to a dramatic increase in costs [5]. On the other hand, early initiation of dalbavancin therapy has been associated with reduced costs due to its potent and rapid bactericidal effects, which may shorten the length of hospital stay [5]. Dalbavancin’s once-weekly dosing regimen allows for OPAT, reducing the burden on healthcare facilities and potentially minimizing overall treatment costs [41]. In a single study, patients with BJI could be discharged 10.6 days earlier, and EUR 3909 could be saved [41]. Another study compared long-acting lipoglycopeptides (LaLGP) with the standard-of-care and showed that the average total healthcare-related cost of care was USD 295,589 in the LaLGP compared to USD 326,089 in the standard-of-care cohort [40]. Moreover, the duration of hospitalization was 22.9 days in the LaLGP group compared to 32 days in the standard-of-care group [40]. Although LaLGP were associated with numerical, though not statistically significant, cost savings and reduced length of stay versus standard of care, the administration of LaLGP may be beneficial for patients with deep-seated, Gram-positive bacterial infections who have socioeconomic factors that preclude oral transition, or OPAT [40]. Finally, another study evaluating the impact of dalbavancin on both hospital length-of-stay and treatment-related costs in a cohort of patients with diverse Gram-positive bacterial infections reported that a median of EUR 8259 and 14 hospital days per patient who received dalbavancin therapy were saved. Hence, the use of dalbavancin provides a significant reduction in both the duration of hospitalization and treatment-related costs [39].

## 4. Clinical Efficacy of Dalbavancin in BJI

### 4.1. Dalbavancin Treatment in Native BJI

#### 4.1.1. Dalbavancin Treatment in Osteomyelitis

Osteomyelitis is a bone infection usually, but not exclusively, caused by Gram-positive pyogenic bacteria, which is associated with serious potential complications and an increased duration of hospitalization [1]. It often requires surgical intervention in addition to prolonged antimicrobial treatment and is considered one of the most challenging infections to treat [1].

To date, several retrospective studies with small sample sizes have been published about dalbavancin’s role in treating osteomyelitis (Table 3) [28,38,42,43,44,45,46,47,48,49,50,51,52,53,54,55,56]. Clinical cure or success was used for the evaluation of the clinical efficacy and outcome of dalbavancin treatment. Clinical success was defined as the resolution of the clinical signs and symptoms compatible with infection without the need for additional debridement, surgical interventions, or antibiotic treatment, as well as, if applicable, the microbiological eradication without the isolation of a pathogen from the cultures. Only one large randomized comparative clinical trial has been published in adults with a first episode of osteomyelitis defined by clinical symptoms, radiological findings, and elevated C-reactive protein (Table 3) [29]. Eighty patients were randomized 7:1 to dalbavancin (1500 mg IV on days 1 and 8) or standard of care treatment for 4–6 weeks, and the clinical response was assessed at 21 days, 6 months, and 1 year. Clinical cure at day 42 was observed in 97% and 88% of the patients in the dalbavancin and standard-of-care treatments, respectively. The clinical success in the patients who received dalbavancin was similar at day 21 (94%), 6 months, and 1 year (96%). This study concluded that a 2-dose regimen of weekly dalbavancin is an effective and safe option for the treatment of osteomyelitis [29]. One retrospective matched cohort study assessed the efficacy of dalbavancin at 3 months after the completion of treatment in 11 patients with osteomyelitis compared to a similar number of patients who received standard-of-care therapy [44]. Clinical success occurred in 100% of the patients in the dalbavancin group and in 82% and 89% in the standard-of-care group at the end of the treatment and at the 3-month follow-up, respectively [44]. No adverse events were noted in either group [44]. In a retrospective, observational cohort study of adult patients with osteomyelitis, patients were matched 1:2 to dalbavancin (a two-dose regimen of 1500 mg 1 week apart) or standard-of-care treatment [48]. A total of 42 patients with osteomyelitis received dalbavancin, and 90 received standard-of-care treatment. Treatment failure was similar between the two treatment groups (21% in the dalbavancin group and 23.3% in the standard-of-care group, respectively) [48]. Patients who received dalbavancin had a shorter hospital stay and fewer adverse events compared with the standard-of-care treatment group. There was no difference in the rate of infection-related readmissions between the two groups [48].

A multicenter retrospective study evaluated the clinical success of dalbavancin in 36 patients with osteomyelitis [49]. Clinical efficacy was achieved in 90% of patients 3 months after the completion of dalbavancin treatment, and no adverse events were reported [49]. Another multicenter retrospective study evaluated the efficacy of dalbavancin use in various Gram-positive infections, and 30 patients with osteomyelitis were included [28]. A two-dose regimen of dalbavancin (1500 mg on days 1 and 8) was shown to have a clinical efficacy of 85% with a good safety and tolerance profile [28]. Other studies assessing the efficacy of dalbavancin in patients with osteomyelitis showed that clinical success rates ranged from 65% to 100%, suggesting that dalbavancin is a suitable and safe option for the treatment of osteomyelitis (Table 3) [38,42,43,45,46,47,50,51,52,53,54,55,56]. Finally, a systematic review and meta-analysis evaluating the efficacy and safety of dalbavancin versus vancomycin in Gram-positive infections showed that dalbavancin was non-inferior to vancomycin for the treatment of osteomyelitis in a phase II trial (OR: 4.64, CI: 0.37–57.93) and was associated with significantly fewer adverse events (OR: 0.73, CI: 0.57–0.94; *p*: 0.01) [57].

Limitations of the studies assessing the efficacy of dalbavancin in the treatment of osteomyelitis include small sample sizes, use of different dosing regimens, short (<1 year) follow-up, as well as heterogeneity of the patients included and anatomical sites of infection.

To date, there is no clinical evidence about the efficacy and safety of dalbavancin in the treatment of osteoarticular infections in the pediatric population. However, a phase 3 multicenter, open-label, comparator-controlled study (NCT02814916) was performed in children with acute bacterial skin and skin structure infections (birth–<18 years old) or sepsis (<3 months old) known/suspected to be caused by susceptible Gram-positive organisms [58]. Children ≥3 months old were randomized 3:3:1 to receive single-dose dalbavancin, 2-dose dalbavancin, or a standard-of-care treatment; those <3 months old received single-dose dalbavancin [58]. Dalbavancin was generally safe and well tolerated and resulted in clinical responses of 97.4% and 98.6% at 48–72 h post-randomization in the single- and 2-dose study arms, respectively. Clinical cure was achieved in >96% of the patients treated with dalbavancin [58]. To conclude, a 2-dose regimen of weekly dalbavancin appears to be a safe and effective option for the management of osteomyelitis in adults. Further clinical studies are required, especially in children who are ideal candidates for antibiotics with prolonged activity.

#### 4.1.2. Dalbavancin Treatment in Septic Arthritis

Septic arthritis is a serious and potentially devastating joint infection that requires prompt and effective antimicrobial therapy [59]. The emergence of multidrug-resistant bacterial strains and the need for prolonged treatment make it crucial to test the use of new antimicrobials that adequately penetrate joints [59]. Clinical evidence supporting the use of dalbavancin in septic arthritis is currently limited, but emerging data from case series and real-world experience show promising results. A favorable clinical outcome with dalbavancin, defined as the improvement or disappearance of all signs and symptoms of infection and discharge from the hospital, was reported in five patients with septic arthritis [43]. In a cohort of patients with BJI, all patients with septic arthritis (*n* = 4) had clinical cure at 6 months follow-up with dalbavancin therapy, while another similar study showed that 75% of patients had clinical success at 6 months after the completion of treatment [42,45].

In a systematic review, 29 patients with septic arthritis treated with dalbavancin were reported, and in 21 cases, the clinical outcome was evaluated. A favorable outcome was observed in 17/21 (80.1%) patients with septic arthritis [60]. A higher percentage of a good response was found in people who received three doses of dalbavancin 1 week apart. In comparison, a higher rate of treatment failure was recorded in cases who received less than two or more than four doses of dalbavancin [60]. Finally, in an ongoing pilot study (NCT03426761), 50 participants with native or prosthetic joint septic arthritis caused by Gram-positive bacteria will be randomized to dalbavancin 1500 mg every 2 weeks or standard of care antimicrobial treatment, and the outcome between the two treatments will be compared [61]. In conclusion, dalbavancin may be a safe therapeutic option for septic arthritis, with good bone and joint penetration, but further evidence is needed to evaluate its efficacy in patients with septic arthritis.

#### 4.1.3. Dalbavancin Treatment in Spondylodiscitis

The efficacy of dalbavancin in spondylodiscitis treatment is not fully established [62], and the available data are limited. One multicenter retrospective study evaluating the efficacy and safety of dalbavancin in 13 patients with spondylodiscitis demonstrated that the drug was well tolerated with minimal adverse effects. Clinical success was achieved in 85% of the patients during hospitalization, while additional antibiotics were required in the remaining two cases [63]. The most frequently used regimen was two weekly doses of 1500 mg, but also one dose of 1000 mg, followed by weekly doses of 500 mg for 6 weeks. Five months after discharge, no deaths were observed, but 42% of the patients required additional antibiotics for signs of infection on follow-up imaging [63]. Other retrospective studies with a small number of patients with spondylodiscitis treated with dalbavancin showed that it is well tolerated with low rates of adverse events, while cure rates varied from 33% to 100% [28,38,42,45,49,56,64,65]. Hence, the results of the efficacy of dalbavancin in spondylodiscitis treatment are variable. Therefore, further studies with a large number of patients and long-term follow-ups are required to evaluate its efficacy in this infection.

#### 4.1.4. Dalbavancin Treatment for Diabetic Foot Infections

Diabetic foot infections represent a serious problem for public health because their consequences may be common and severe, such as osteomyelitis and amputation [66]. The management of diabetic foot osteomyelitis requires prolonged antimicrobial treatment for Gram-positive bacteria [66]. One study evaluated the in vitro activity of dalbavancin against a panel of Gram-positive bacterial strains isolated from bone biopsies of patients with suspected diabetic foot osteomyelitis and showed excellent activity against all Gram-positive pathogens [67]. With MIC50 and MIC90 values of 0.047 and 0.094 mg/L, respectively, dalbavancin showed the most potent in vitro anti-biofilm activity among the antimicrobial agents tested [67]. Clinical evidence supporting the use of dalbavancin in diabetic foot infections is currently limited, but data from one retrospective study of 23 patients showed favorable clinical outcomes and reduced length of hospital stay [68]. The median duration of treatment was 5 weeks, and the most commonly used regimen of dalbavancin was 1000 mg, followed by 500 mg weekly for 5 weeks. Mild side effects, such as nausea and gastrointestinal discomfort, were reported in only three patients [68]. At 90 days after completion of dalbavancin therapy, 87% of the patients were cured [68]. Finally, one case of diabetic foot osteomyelitis caused by multidrug-resistant *Enterococcus faecium* received two doses of 1500 mg dalbavancin followed by oral linezolid with clinical improvement. These data suggest that dalbavancin could be a safe alternative for treating deep diabetic foot infections [69]. However, further research, especially randomized clinical trials, is certainly needed to establish the optimal role of dalbavancin in the management of diabetic foot osteomyelitis.

### 4.2. Dalbavancin Treatment in PJI

PJI is a challenging complication after hip and knee replacement that can lead to multiple revision surgeries, prolonged hospitalization, and increased morbidity and mortality [70]. These infections are complex because the formation of biofilms enables bacteria to evade the host immune system and results in antimicrobial resistance and infection persistence [70]. The clinical data about dalbavancin treatment for PJI are scarce and heterogeneous, but its use is an option in the setting of prosthesis removal. In a cohort study, 89 patients with PJI who received at least two doses of dalbavancin were compared to 89 patients with PJI who were treated with standard-of-care [71]. Infection eradication and re-implantation rates were similar between the two groups, but there were significantly fewer Gram-positive bacteria detected in culture-positive re-revisions in the patients treated with dalbavancin [71]. In another retrospective study, which included 16 patients with PJI treated with dalbavancin, infection resolved in 12 and treatment failed in only 2 [72], while in one retrospective study with eight patients with PJI, clinical cure at 6-month follow-up was noted in 75% of the patients treated with a combination of dalbavancin and surgical intervention [42]. The efficacy of dalbavancin was evaluated retrospectively in a cohort of 17 patients with PJI, and a clinical cure was achieved in 47.1% of the patients, which is lower than usually reported. This may have happened because dalbavancin was mostly used as salvage therapy in complicated polymicrobial infections [73]. In a review of all cases of PJI treated with dalbavancin available in the literature, the overall clinical cure was estimated at 73.1% [73]. It is worth noting that most of the cases (75%) were treated with prosthesis removal along with dalbavancin therapy. In 92.6% of them, a clinical cure was observed after a median of 16 months, while 19.4% of the cases were treated with prosthesis retention plus dalbavancin, and in 42.9% of them, successful retention of the prosthesis in the short term was achieved [72]. In addition, a retrospective multicenter study, which included only two patients with PJI, showed that dalbavancin may be used as suppressive antibiotic therapy in these infections, as clinical efficacy was observed in one of two patients and no adverse events were reported [74]. Finally, several in vitro and experimental in vivo studies demonstrate that dalbavancin successfully eliminates biofilms due to Gram-positive bacteria, indicating that it is an active therapy against biofilms [75,76,77,78,79,80,81].

## 5. Conclusions

Dalbavancin represents a promising treatment option for BJI, offering potent activity against key pathogens and an extended dosing interval that may enhance patient adherence and shorten the duration of hospitalization. Although the cost of a single infusion of dalbavancin is substantial, eliminating the need for a long-term intravenous catheter with its demanding daily maintenance will make outpatient treatment of BJI attractive and cost-effective. Clinical studies have shown non-inferiority to standard-of-care therapies, supporting their use in managing these challenging infections. Moreover, dalbavancin’s favorable safety profile and lack of drug–drug interactions add to its appeal as an alternative option to traditional antibiotic regimens. Further research in adults, especially children, who have difficult intravenous access and are ideal candidates for long-acting antibiotics, is necessary to evaluate the role of dalbavancin in the therapeutic armamentarium of BJI.

## Figures and Tables

**Figure 3:**
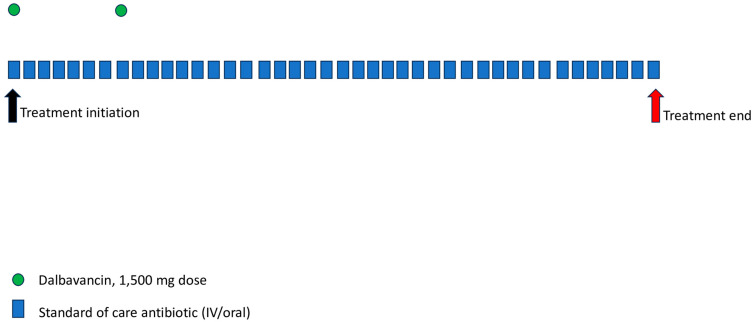
Schematic representation of dalbavancin versus standard of care antibiotic therapy for BJI. Each blue box represents one day of therapy.

**Table 1:** Proposed regimens of dalbavancin in adults and children for the treatment of BJI.

Dose Regimens of Dalbavancin in BJI	
Adults	a. 1500 mg administered on days 1 and 8 (Figure 3)
	b. 1000 mg initially, followed by 4 subsequent weekly doses of 500 mg
Children	a. 3 months to <6 years: 22.5 mg/kg (1500 mg maximum) on days 1 and 8
	b. 3 months to <6 years: 15 mg/kg (1000 mg maximum) on day 1 and 7.5 mg/kg (500 mg maximum) on day 8
	a. 6 to <18 years: 18 mg/kg (1500 mg maximum) on days 1 and 8
	b. 6 to <18 years: 12 mg/kg (1000 mg maximum) on day 1 and 6 mg/kg (500 mg maximum) on day 8

**Table 2:** Common adverse events of dalbavancin.

Nausea Vomiting Abdominal pain Headache Rash Pruritus Urinary tract infections Constipation Diarrhea

**Table 3:** Studies assessing dalbavancin’s effectiveness and safety in patients with osteomyelitis.

Authors (Year) [Ref]	Design	Sample Size	Dalbavancin Regimen	Follow-Up	Clinical Outcome	Adverse Events
**Bouza et al. (2018) [39]**	Retrospective	12	2 doses of 1500 mg 1 week apart, or 1000 mg × 1, followed by 500 mg weekly Median duration: 3 weeks (range 1–24)	≥1 month	Clinical success: 92%	AE in 13% of the patients
**Nunez-Nunez et al. (2018) [44]**	Prospective observational	6	2 doses of 1500 mg weekly, or 1000 mg × 1, followed by 500 smg weekly	3 months	Clinical success: 100%	Mild AE in 4.5% of the patients
**Rappo et al. (2019) [20]**	Prospective, RCT	80 Dalbavancin: 70 vs. SOC: 10	2 doses of 1500 mg 1 week apart	12 months	Clinical cure: 94% at day 21, 97% at day 42 and 96% at 6 months and 1 year	Treatment-emergent AE in 10 (14.3%) patients
**Tobudic et al. (2019) [19]**	Retrospective	20	Various regimens: 1500 mg × 1, followed by 1000 mg every 2 weeks, or 1000 mg × 1, followed by 500 mg weekly, or 2 doses of 1500 mg 1 week apart Median duration: 8 weeks (range 4–32)	6 months	Clinical cure: 60%	Mild AE, such as exanthema, nausea, and hyperglycaemia, in 5% of the patients
**Wunsch et al. (2019) [19]**	Retrospective	30	Various regimens: 1500 mg weekly, or 1000 mg × 1, followed by 500 mg weekly Median doses: 3 (range 1–32)	3 months	Clinical success: 89%	Mild AE in 3% of the patients
**Almangour et al. (2019) [41]**	Retrospective	31	Various regimens: 1500 mg weekly, or 1000 mg × 1, followed by 500 mg weekly Median doses: 3 (range 1–14)	3 months	Clinical cure: 90%	None
**Morata et al. (2019) [30]**	Retrospective	19	Various regimens: 1500 mg weekly, or 1000 mg × 1, followed by 500 mg weekly, or 1000 mg weekly Median doses: 2 (IQR 2–4)	Median: 164 days (IQR:93–262.5)	Clinical success: 89.5%	Mild AE in 7 patients
**Morrisette et al. (2019) [42]**	Retrospective	15	NA	Median: 6.1 months (IQR: 3.7–11.8)	Clinical success: 92%	Mild AE in 11% of the patients
**Bryson-Cahn et al. (2019) [48]**	Retrospective	7	Various regimens: 1500 mg weekly, or 1000 mg × 1, followed by 500 mg weekly, or 1000 mg weekly Median doses: 1 (IQR 1–5)	1–12 months	Clinical cure: 71.4%	None
**Bork et al. (2019) [46]**	Retrospective	13	NA Median doses: 3 (IQR 4.5)	3 months	Clinical success: 46% at 30 days	1 patient with generalized pruritus and rash and 1 patient with acute kidney injury
**Dinh et al. (2019) [45]**	Retrospective	48	Various regimens: 2 doses of 1500 mg weekly, or 1500 mg every 2 weeks, or 1000 mg × 1, followed by 500 mg weekly/every 2 weeks Median duration: 14 days (IQR: 14–19.25)	Mean: 87.8 ± 86.9 days	Clinical cure: 76.1%	Mild AE in 5 patients
**Bartoletti et al. (2019) [47]**	Retrospective	15	2 doses of 1500 mg weekly, or 1000 mg × 1, followed by 500 mg weekly Median doses: 4	6 months	Clinical cure: 93% at 6 months	None
**Bai et al. (2020) [37]**	Retrospective	29	1500 mg weekly Maximum 7 doses	1–3 months	Clinical cure: 89.7%	Mild AE in 5.4% of the patients
**Almangour et al. (2020) [36]**	Retrospective Matched cohort study	21 Dalbavancin:11 vs. SOC:10	Various regimens: 1500 mg weekly, or 1000 mg × 1, followed by 500 mg weekly Median duration: 42 days (IQR 5)	3 months	Clinical cure: 100%	None
**Vazquez Deida et al. (2020) [43]**	Retrospective	5	1500 mg × 1	3 months	Clinical cure: 80%	Mild AE in 7% of the patients
**Brescini et al. (2021) [35]**	Retrospective	8	Various regimens: 2 doses of 1500 mg weekly, or 1500 mg every 2 weeks Median doses: 1 (IQR: 1–9)	1–3 months	Clinical success: 100%	Rash in 1 patient
**Cain et al. (2021) [40]**	Retrospective	132 Dalbavancin: 42 vs. SOC: 90	2 doses of 1500 mg weekly	12 months	Clinical cure: 78.6%	Mild AE in 21.4% of the patients
**Taylor et al. (2022) [38]**		26	1500 mg weekly Range: 1–4 doses	3 months	Clinical success: 87%	None

AE: Adverse events; IQR: Interquartile range; NA: Non-applicable; RCT: Randomized controlled trial; SOC: Standard-of-care treatment.

## Data Availability

The data underlying this article are available from the corresponding author upon reasonable request.

## References

[1] Lew P.D.P., Waldvogel P.F.A. (2004). Osteomyelitis. Lancet.

[2] Colston J., Atkins B. (2018). Bone and Joint Infection. Clin. Med..

[3] Mathews C.J., Holloway A.M. (2022). Bone and Joint Infections. Medicine.

[4] Darton T., Townsend R. (2010). Bone and Joint Infections. Surgery.

[5] Darley E.S.R., MacGowan A.P. (2004). Antibiotic Treatment of Gram-Positive Bone and Joint Infections. J. Antimicrob. Chemother..

[6] Woods C.R., Bradley J.S., Chatterjee A., Copley L.A., Robinson J., Kronman M.P., Arrieta A., Fowler S.L., Harrison C., Carrillo-Marquez M.A. (2021). Clinical Practice Guideline by the Pediatric Infectious Diseases Society and the Infectious Diseases Society of America: 2021 Guideline on Diagnosis and Management of Acute Hematogenous Osteomyelitis in Pediatrics. J. Pediatr. Infect. Dis. Soc..

[7] Berbari E.F., Kanj S.S., Kowalski T.J., Darouiche R.O., Widmer A.F., Schmitt S.K., Hendershot E.F., Holtom P.D., Huddleston P.M., Petermann G.W. (2015). 2015 Infectious Diseases Society of America (IDSA) Clinical Practice Guidelines for the Diagnosis and Treatment of Native Vertebral Osteomyelitis in Adults. Clin. Infect. Dis..

[8] Liu C., Bayer A., Cosgrove S.E., Daum R.S., Fridkin S.K., Gorwitz R.J., Kaplan S.L., Karchmer A.W., Levine D.P., Murray B.E. (2011). Clinical Practice Guidelines by the Infectious Diseases Society of America for the Treatment of Methicillin-Resistant Staphylococcus Aureus Infections in Adults and Children. Clin. Infect. Dis..

[9] Bernard L., Arvieux C., Brunschweiler B., Touchais S., Ansart S., Bru J.-P., Oziol E., Boeri C., Gras G., Druon J. (2021). Antibiotic Therapy for 6 or 12 Weeks for Prosthetic Joint Infection. N. Engl. J. Med..

[10] Lodise T.P., McKinnon P.S. (2007). Burden of Methicillin-Resistant Staphylococcus Aureus: Focus on Clinical and Economic Outcomes. Pharmacotherapy.

[11] Styers D., Sheehan D.J., Hogan P., Sahm D.F. (2006). Laboratory-Based Surveillance of Current Antimicrobial Resistance Patterns and Trends among Staphylococcus Aureus: 2005 Status in the United States. Ann. Clin. Microbiol. Antimicrob..

[12] Antunes A.L.S., Bonfanti J.W., Perez L.R.R., Pinto C.C.F., de Freitas A.L.P., Macedo A.J., Barth A.L. (2011). High Vancomycin Resistance among Biofilms Produced by Staphylococcus Species Isolated from Central Venous Catheters. Mem. Inst. Oswaldo Cruz.

[13] Limbago B.M., Kallen A.J., Zhu W., Eggers P., McDougal L.K., Albrecht V.S. (2014). Report of the 13th Vancomycin-Resistant Staphylococcus Aureus Isolate from the United States. J. Clin. Microbiol..

[14] Steenbergen J.N., Alder J., Thorne G.M., Tally F.P. (2005). Daptomycin: A Lipopeptide Antibiotic for the Treatment of Serious Gram-Positive Infections. J. Antimicrob. Chemother..

[15] Cave K., Gould I. (2022). Daptomycin. Compr. Pharmacol..

[16] Diekema D.J., Jones R.N. (2001). Oxazolidinone Antibiotics. Lancet.

[17] Azzouz A., Preuss C.V. (2023). Linezolid.

[18] Klinker K.P., Borgert S.J. (2015). Beyond Vancomycin: The Tail of the Lipoglycopeptides. Clin. Ther..

[19] Cooper C.C., Stein G.E., Mitra S., Abubaker A., Havlichek D.H. (2021). Long-Acting Lipoglycopeptides for the Treatment of Bone and Joint Infections. Surg. Infect..

[20] Gonzalez D., Bradley J.S., Blumer J., Yogev R., Watt K.M., James L.P., Palazzi D.L., Bhatt-Mehta V., Sullivan J.E., Zhang L. (2017). Dalbavancin Pharmacokinetics and Safety in Children 3 Months to 11 Years of Age. Pediatr. Infect. Dis. J..

[21] Dunne M.W., Puttagunta S., Giordano P., Krievins D., Zelasky M., Baldassarre J. (2016). A Randomized Clinical Trial of Single-Dose Versus Weekly Dalbavancin for Treatment of Acute Bacterial Skin and Skin Structure Infection. Clin. Infect. Dis..

[22] Wang Y., Wang J., Wang R., Li Y., Cai Y. (2021). Efficacy and Safety of Dalbavancin in the Treatment of Gram-Positive Bacterial Infections. J. Glob. Antimicrob. Resist..

[23] Bailey J., Summers K.M. (2008). Dalbavancin: A New Lipoglycopeptide Antibiotic. Am. J. Health Syst. Pharm..

[24] Dash R.P., Babu R.J., Srinivas N.R. (2017). Review of the Pharmacokinetics of Dalbavancin, a Recently Approved Lipoglycopeptide Antibiotic. Infect. Dis..

[25] Marbury T., Dowell J.A., Seltzer E., Buckwalter M. (2009). Pharmacokinetics of Dalbavancin in Patients with Renal or Hepatic Impairment. J. Clin. Pharmacol..

[26] Andes D., Craig W.A. (2007). In Vivo Pharmacodynamic Activity of the Glycopeptide Dalbavancin. Antimicrob. Agents Chemother..

[27] Dunne M.W., Puttagunta S., Sprenger C.R., Rubino C., Van Wart S., Baldassarre J. (2015). Extended-Duration Dosing and Distribution of Dalbavancin into Bone and Articular Tissue. Antimicrob. Agents Chemother..

[28] Wunsch S., Krause R., Valentin T., Prattes J., Janata O., Lenger A., Bellmann-Weiler R., Weiss G., Zollner-Schwetz I. (2019). Multicenter Clinical Experience of Real Life Dalbavancin Use in Gram-Positive Infections. Int. J. Infect. Dis..

[29] Rappo U., Puttagunta S., Shevchenko V., Shevchenko A., Jandourek A., Gonzalez P.L., Suen A., Mas Casullo V., Melnick D., Miceli R. (2019). Dalbavancin for the Treatment of Osteomyelitis in Adult Patients: A Randomized Clinical Trial of Efficacy and Safety. Open Forum Infect. Dis..

[30] Bradley J.S., Puttagunta S., Rubino C.M., Blumer J.L., Dunne M., Sullivan J.E. (2015). Pharmacokinetics, Safety and Tolerability of Single Dose Dalbavancin in Children 12–17 Years of Age. Pediatr. Infect. Dis. J..

[31] Pfaller M.A., Flamm R.K., Castanheira M., Sader H.S., Mendes R.E. (2018). Dalbavancin In-Vitro Activity Obtained against Gram-Positive Clinical Isolates Causing Bone and Joint Infections in US and European Hospitals (2011–2016). Int. J. Antimicrob. Agents.

[32] Pfaller M.A., Mendes R.E., Duncan L.R., Flamm R.K., Sader H.S. (2018). Activity of Dalbavancin and Comparator Agents against Gram-Positive Cocci from Clinical Infections in the USA and Europe 2015–2016. J. Antimicrob. Chemother..

[33] Silva V., Antão H.S., Guimarães J., Prada J., Pires I., Martins Â., Maltez L., Pereira J.E., Capelo J.L., Igrejas G. (2020). Efficacy of Dalbavancin against MRSA Biofilms in a Rat Model of Orthopaedic Implant-Associated Infection. J. Antimicrob. Chemother..

[34] Silva V., Miranda C., Bezerra M., Antão H.S., Guimarães J., Prada J., Pires I., Maltez L., Pereira J.E., Capelo J.L. (2021). Anti-Biofilm Activity of Dalbavancin against Methicillin-Resistant Staphylococcus Aureus (MRSA) Isolated from Human Bone Infection. J. Chemother..

[35] McCurdy S.P., Jones R.N., Mendes R.E., Puttagunta S., Dunne M.W. (2015). In Vitro Activity of Dalbavancin against Drug-Resistant Staphylococcus Aureus Isolates from a Global Surveillance Program. Antimicrob. Agents Chemother..

[36] Dunne M.W., Talbot G.H., Boucher H.W., Wilcox M., Puttagunta S. (2016). Safety of Dalbavancin in the Treatment of Skin and Skin Structure Infections: A Pooled Analysis of Randomized, Comparative Studies. Drug Saf..

[37] Boucher H.W., Wilcox M., Talbot G.H., Puttagunta S., Das A.F., Dunne M.W. (2014). Once-Weekly Dalbavancin versus Daily Conventional Therapy for Skin Infection. N. Engl. J. Med..

[38] Morata L., Cobo J., Fernández-Sampedro M., Guisado Vasco P., Ruano E., Lora-Tamayo J., Sánchez Somolinos M., González Ruano P., Rico Nieto A., Arnaiz A. (2019). Safety and Efficacy of Prolonged Use of Dalbavancin in Bone and Joint Infections. Antimicrob. Agents Chemother..

[39] Poliseno M., Bavaro D.F., Brindicci G., Luzzi G., Carretta D.M., Spinarelli A., Messina R., Miolla M.P., Achille T.I., Dibartolomeo M.R. (2021). Dalbavancin Efficacy and Impact on Hospital Length-of-Stay and Treatment Costs in Different Gram-Positive Bacterial Infections. Clin. Drug Investig..

[40] Antosz K., Al-Hasan M.N., Lu Z.K., Tabor B., Justo J.A., Milgrom A., Kohn J., Bookstaver P.B. (2021). Clinical Utility and Cost Effectiveness of Long-Acting Lipoglycopeptides Used in Deep-Seated Infections among Patients with Social and Economic Barriers to Care. Pharmacy.

[41] Wilke M., Worf K., Preisendörfer B., Heinlein W., Kast T., Bodmann K.-F. (2019). Potential Savings through Single-Dose Intravenous Dalbavancin in Long-Term MRSA Infection Treatment—A Health Economic Analysis Using German DRG Data. GMS Infect. Dis..

[42] Tobudic S., Forstner C., Burgmann H., Lagler H., Steininger C., Traby L., Vossen M.G., Winkler S., Thalhammer F. (2019). Real-World Experience with Dalbavancin Therapy in Gram-Positive Skin and Soft Tissue Infection, Bone and Joint Infection. Infection.

[43] Brescini L., Della Martera F., Morroni G., Mazzanti S., Di Pietrantonio M., Mantini P., Candelaresi B., Pallotta F., Olivieri S., Iencinella V. (2021). Use of Dalbavancin in Skin, Bone and Joint Infections: A Real-Life Experience in an Italian Center. Antibiotics.

[44] Almangour T.A., Perry G.K., Alhifany A.A. (2020). Dalbavancin versus Standard of Care for the Treatment of Osteomyelitis in Adults: A Retrospective Matched Cohort Study. Saudi Pharm. J..

[45] Bai F., Aldieri C., Cattelan A.M., Raumer F., Di Meco E., Moioli M.C., Tordato F., Morelli P., Borghi F., Rizzi M. (2020). Efficacy and Safety of Dalbavancin in the Treatment of Acute Bacterial Skin and Skin Structure Infections (ABSSSIs) and Other Infections in a Real-Life Setting: Data from an Italian Observational Multicentric Study (DALBITA Study). Expert. Rev. Anti. Infect. Ther..

[46] Taylor K., Williamson J., Luther V., Stone T., Johnson J., Gruss Z., Russ-Friedman C., Ohl C., Beardsley J. (2022). Evaluating the Use of Dalbavancin for Off-Label Indications. Infect. Dis. Rep..

[47] Bouza E., Valerio M., Soriano A., Morata L., Carus E.G., Rodríguez-González C., Hidalgo-Tenorio M.C., Plata A., Muñoz P., Vena A. (2018). Dalbavancin in the Treatment of Different Gram-Positive Infections: A Real-Life Experience. Int. J. Antimicrob. Agents.

[48] Cain A.R., Bremmer D.N., Carr D.R., Buchanan C., Jacobs M., Walsh T.L., Moffa M.A., Shively N.R., Trienski T.L. (2022). Effectiveness of Dalbavancin Compared With Standard of Care for the Treatment of Osteomyelitis: A Real-World Analysis. Open Forum Infect. Dis..

[49] Almangour T.A., Perry G.K., Terriff C.M., Alhifany A.A., Kaye K.S. (2019). Dalbavancin for the Management of Gram-Positive Osteomyelitis: Effectiveness and Potential Utility. Diagn. Microbiol. Infect. Dis..

[50] Morrisette T., Miller M.A., Montague B.T., Barber G.R., Brett McQueen R., Krsak M. (2019). On- And off-Label Utilization of Dalbavancin and Oritavancin for Gram-Positive Infections. J. Antimicrob. Chemother..

[51] Vazquez Deida A.A., Shihadeh K.C., Preslaski C.R., Young H.L., Wyles D.L., Jenkins T.C. (2020). Use of a Standardized Dalbavancin Approach to Facilitate Earlier Hospital Discharge for Vulnerable Patients Receiving Prolonged Inpatient Antibiotic Therapy. Open Forum Infect. Dis..

[52] Núñez-Núñez M., Casas-Hidalgo I., García-Fumero R., Vallejo-Rodríguez I., Anguita-Santos F., Hernández-Quero J., Cabeza-Barrera J., Ruiz-Sancho A. (2020). Dalbavancin Is a Novel Antimicrobial against Gram-Positive Pathogens: Clinical Experience beyond Labelled Indications. Eur. J. Hosp. Pharm..

[53] Dinh A., Duran C., Pavese P., Khatchatourian L., Monnin B., Bleibtreu A., Denis E., Etienne C., Rouanes N., Mahieu R. (2019). French National Cohort of First Use of Dalbavancin: A High Proportion of off-Label Use. Int. J. Antimicrob. Agents.

[54] Bork J.T., Heil E.L., Berry S., Lopes E., Davé R., Gilliam B.L., Amoroso A. (2019). Dalbavancin Use in Vulnerable Patients Receiving Outpatient Parenteral Antibiotic Therapy for Invasive Gram-Positive Infections. Infect. Dis. Ther..

[55] Bartoletti M., Mikus E., Pascale R., Giannella M., Tedeschi S., Calvi S., Tenti E., Tumietto F., Viale P. (2019). Clinical Experience with Dalbavancin for the Treatment of Deep Sternal Wound Infection. J. Glob. Antimicrob. Resist..

[56] Bryson-Cahn C., Beieler A.M., Chan J.D., Harrington R.D., Dhanireddy S. (2019). Dalbavancin as Secondary Therapy for Serious Staphylococcus Aureus Infections in a Vulnerable Patient Population. Open Forum Infect. Dis..

[57] Jame W., Basgut B., Abdi A. (2021). Efficacy and Safety of Novel Glycopeptides versus Vancomycin for the Treatment of Gram-Positive Bacterial Infections Including Methicillin Resistant Staphylococcus Aureus: A Systematic Review and Meta-Analysis. PLoS ONE.

[58] Giorgobiani M., Burroughs M.H., Antadze T., Carrothers T.J., Riccobene T.A., Patel R., Lin T., Stefanova P. (2023). The Safety and Efficacy of Dalbavancin and Active Comparator in Pediatric Patients With Acute Bacterial Skin and Skin Structure Infections. Pediatr. Infect. Dis. J..

[59] Castellazzi L., Mantero M., Esposito S. (2016). Update on the Management of Pediatric Acute Osteomyelitis and Septic Arthritis. Int. J. Mol. Sci..

[60] Lovatti S., Tiecco G., Mulé A., Rossi L., Sforza A., Salvi M., Signorini L., Castelli F., Quiros-Roldan E. (2023). Dalbavancin in Bone and Joint Infections: A Systematic Review. Pharmaceuticals.

[61] Cooper M.M., Preslaski C.R., Shihadeh K.C., Hawkins K.L., Jenkins T.C. (2021). Multiple-Dose Dalbavancin Regimens as the Predominant Treatment of Deep-Seated or Endovascular Infections: A Scoping Review. Open Forum Infect. Dis..

[62] Andreoni M., Bassetti M., Corrao S., De Rosa F.G., Esposito V., Falcone M., Grossi P., Pea F., Petrosillo N., Tascini C. (2021). The Role of Dalbavancin for Gram Positive Infections in the COVID-19 Era: State of the Art and Future Perspectives. Expert. Rev. Anti. Infect. Ther..

[63] Ramadan M.S., Gallo R., Lugarà M., Gambardella M., Oliva G., Bertolino L., Andini R., Coppola N., Zampino R., Durante-Mangoni E. (2022). Dalbavancin Treatment for Spondylodiscitis: Multi-Center Clinical Experience and Literature Review. J. Chemother..

[64] Veve M.P., Patel N., Smith Z.A., Yeager S.D., Wright L.R., Shorman M.A. (2020). Comparison of Dalbavancin to Standard-of-Care for Outpatient Treatment of Invasive Gram-Positive Infections. Int. J. Antimicrob. Agents.

[65] Almangour T.A., Fletcher V., Alessa M., Alhifany A.A., Tabb D. (2017). Multiple Weekly Dalbavancin Dosing for the Treatment of Native Vertebral Osteomyelitis Caused by Methicillin-Resistant Staphylococcus Aureus: A Case Report. Am. J. Case Rep..

[66] Pitocco D., Spanu T., Di Leo M., Vitiello R., Rizzi A., Tartaglione L., Fiori B., Caputo S., Tinelli G., Zaccardi F. (2019). Diabetic Foot Infections: A Comprehensive Overview. Eur. Rev. Med. Pharmacol. Sci..

[67] Pantel A., Nachar O., Boudet A., Loubet P., Schuldiner S., Cellier N., Sotto A., Dunyach-Remy C., Lavigne J.P. (2021). In Vitro Activity of Dalbavancin against Gram-Positive Bacteria Isolated from Diabetic Foot Osteomyelitis. J. Antimicrob. Chemother..

[68] Navarro-Jiménez G., Fuentes-Santos C., Moreno-Núñez L., Alfayate-García J., Campelo-Gutierrez C., Sanz-Márquez S., Pérez-Fernández E., Velasco-Arribas M., Hervás-Gómez R., Martín-Segarra O. (2022). Experience in the Use of Dalbavancin in Diabetic Foot Infection. Enfermedades Infecc. Microbiol. Clin..

[69] Loupa C.V., Lykoudi E., Meimeti E., Moisoglou I., Voyatzoglou E.D., Kalantzi S., Konsta E. (2020). Successful Treatment of Diabetic Foot Osteomyelitis with Dalbavancin. Med. Arch..

[70] Parvizi J., Tan T.L., Goswami K., Higuera C., Della Valle C., Chen A.F., Shohat N. (2018). The 2018 Definition of Periprosthetic Hip and Knee Infection: An Evidence-Based and Validated Criteria. J Arthroplast..

[71] Simon S., Frank B.J.H., Hartmann S., Hinterhuber L., Reitsamer M., Aichmair A., Dominkus M., Söderquist B., Hofstaetter J.G. (2022). Dalbavancin in Gram-Positive Periprosthetic Joint Infections. J. Antimicrob. Chemother..

[72] Buzón Martín L., Mora Fernández M., Perales Ruiz J.M., Ortega Lafont M., Álvarez Paredes L., Morán Rodríguez M.A., Fernández Regueras M., Machín Morón M.A., Mejías Lobón G. (2019). Dalbavancin for Treating Prosthetic Joint Infections Caused by Gram-Positive Bacteria: A Proposal for a Low Dose Strategy. A Retrospective Cohort Study. Rev. Esp. Quim..

[73] Matt M., Duran C., Courjon J., Lotte R., Moing V.L., Monnin B., Pavese P., Chavanet P., Khatchatourian L., Tattevin P. (2021). Dalbavancin Treatment for Prosthetic Joint Infections in Real-Life: A National Cohort Study and Literature Review. J. Glob. Antimicrob. Resist..

[74] Ruiz-Sancho A., Núñez-Núñez M., Castelo-Corral L., Martínez-Marcos F.J., Lois-Martínez N., Abdul-Aziz M.H., Vinuesa-García D. (2023). Dalbavancin as Suppressive Antibiotic Therapy in Patients with Prosthetic Infections: Efficacy and Safety. Front. Pharmacol..

[75] Baldoni D., Furustrand Tafin U., Aeppli S., Angevaare E., Oliva A., Haschke M., Zimmerli W., Trampuz A. (2013). Activity of Dalbavancin, Alone and in Combination with Rifampicin, against Meticillin-Resistant Staphylococcus Aureus in a Foreign-Body Infection Model. Int. J. Antimicrob. Agents.

[76] Knafl D., Tobudic S., Cheng S.C., Bellamy D.R., Thalhammer F. (2017). Dalbavancin Reduces Biofilms of Methicillin-Resistant Staphylococcus Aureus (MRSA) and Methicillin-Resistant Staphylococcus Epidermidis (MRSE). Eur. J. Clin. Microbiol. Infect. Dis..

[77] Žiemytė M., Rodríguez-Díaz J.C., Ventero M.P., Mira A., Ferrer M.D. (2020). Effect of Dalbavancin on Staphylococcal Biofilms When Administered Alone or in Combination With Biofilm-Detaching Compounds. Front. Microbiol..

[78] Barnea Y., Lerner A., Aizic A., Navon-Venezia S., Rachi E., Dunne M.W., Puttagunta S., Carmeli Y. (2016). Efficacy of Dalbavancin in the Treatment of MRSA Rat Sternal Osteomyelitis with Mediastinitis. J. Antimicrob. Chemother..

[79] Di Pilato V., Ceccherini F., Sennati S., D’Agostino F., Arena F., D’Atanasio N., Di Giorgio F.P., Tongiani S., Pallecchi L., Rossolini G.M. (2020). In Vitro Time-Kill Kinetics of Dalbavancin against Staphylococcus Spp. Biofilms over Prolonged Exposure Times. Diagn. Microbiol. Infect. Dis..

[80] Darouiche R.O., Mansouri M.D. (2005). Dalbavancin Compared with Vancomycin for Prevention of Staphylococcus Aureus Colonization of Devices in Vivo. J. Infect..

[81] Sánchez-Somolinos M., Díaz-Navarro M., Benjumea A., Tormo M., Matas J., Vaquero J., Muñoz P., Sanz-Ruíz P., Guembe M. (2022). Determination of the Elution Capacity of Dalbavancin in Bone Cements: New Alternative for the Treatment of Biofilm-Related Peri-Prosthetic Joint Infections Based on an In Vitro Study. Antibiotics.

